# Supplementary material for: Crystallographic and kinetic analyses of human IPMK reveal disordered domains modulate ATP binding and kinase activity
Source: Sci Rep. 2018 Nov 12;8:16672. doi: 10.1038/s41598-018-34941-3 (PMC6232094; doi:10.1038/s41598-018-34941-3)
Supplement: Supplementary file 1 — Supplementary Information [file 41598_2018_34941_MOESM1_ESM.pdf]

Supplementary Information

Title: Crystallographic and kinetic analyses of human IPMK reveal disordered domains modulate ATP binding and kinase activity.

Authors: Corey D. Seacrist and Raymond D. Blind

```
hIPMK      MATEPPSPLRVEAPGPPEMRTSPAIESTPEGTPQPAGGRLRFLNGCVPLSHQVAGHMYGK   60
ΔIPMK      ------------------------------------------------------MHHHHH   6

hIPMK      DKVGILQHPDGTVLKQLQPPPRGPRELEFYNMVYAADCFDGVLLELRKYLPKYYGIWSPP   120
ΔIPMK      HENLYFQGMDGTVLKQLQPPPRGPRELEFYNMVYAADCFDGVLLELRKYLPKYYGIWSPP   66
                    **************************************************

hIPMK      TAPNDLYLKLEDVTHKFNKPCIMDVKIGQKSYDPFASSEKIQQQVSKYPLMEEIGFLVLG   180
ΔIPMK      TAPNDLYLKLEDVTHKFNKPCIMDVKIGQKSYDPFASSEKIQQQVSKYPLMEEIGFLVLG   126
           ************************************************************

hIPMK      MRVYHVHSDSYETENQHYGRSLTKETIKDGVSRFFHNGYCLRKDAVAASIQKIEKILQWF   240
ΔIPMK      MRVYHVHSDSYETENQHYGRSLTKETIKDGVSRFFHNGYCLRKDAVAASIQKIEKILQWF   186
           ************************************************************

hIPMK      ENQKQLNFYASSLLFVYEGSSQPTTTKLNDRTLAEKFLSKGQLSDTEVLEYNNNFHVLSS   300
ΔIPMK      ENQKQLNFYASSLLFVYEGSSQPTTTKLNDRTLAEKFLSGGGGSG---------------   231
           ***************************************

hIPMK      TANGKIESSVGKSLSKMYARHRKIYTKKHHSQTSLKVENLEQDNGWKSMSQEHLNGNVLS   360
ΔIPMK      ------------------------------------------------------------   231

hIPMK      QLEKVFYHLPTGCQEIAEVEVRMIDFAHVFPSNTIDEGYVYGLKHLISVLRSILDN       416
ΔIPMK      ---------GGGSQEIAEVEVRMIDFAHVFPSNTIDEGYVYGLKHLISVLRSILDN       278
                      *******************************************
```

Figure S1. Sequence alignment of full length human IPMK and ΔIPMK. ΔIPMK does not possess residues 1-69 and 279-373 of the full-length human IPMK sequence. Red represents catalytic regions, blue the IP-helices that bind substrate and green an artificial linker sequence added to maintain protein stability. The green artificial $(Gly_4\text{-}Ser)_2$ linker was inserted between residues 279 and 373.

Table S1. Human IPMK construct expression in bacteria and crystallization.

N-term Domain Boundary	Internal-Loop Boundaries	Tags	Internal-Loop Linker	Relative Expression	Crystals
D70	S279-C373	N-term 6XHis	(GGGGS)2	+++	Yes
G37	S279-C373	C-term 6XHis	(GGGGS)2	+	None
M1	Q262-P370	N-term 6XHis-MBP	None	-	n.d.
G59	Q262-P370	N-term 6XHis MBP	None	-	n.d.

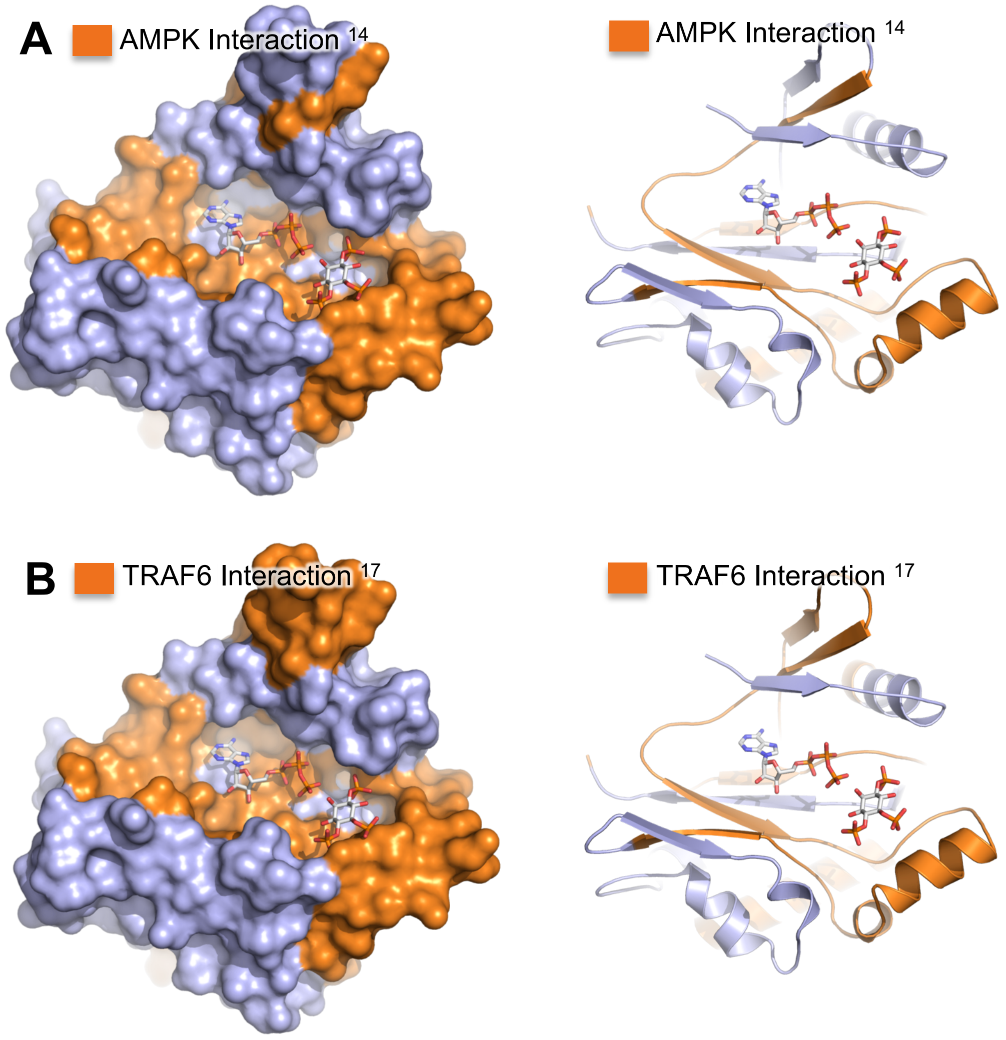

Figure S2. Protein-protein interaction sites superimpose onto IPMK ATP- and substrate-binding sites. Surface (left) and ribbon (right) representations of ΔIPMK, with ADP and PIP_2 modeled into the structure. **A.** IPMK interaction site with AMPK has been mapped[14] to IPMK exons 4 and 6, depicted as orange. With the remainder of the IPMK kinase domain is depicted in light blue. Both ADP and an inositol phosphate kinase substrate were modeled into the structure for reference. **C.** IPMK interaction site with TRAF6 has been mapped[17] to exons 4 and 6, depicted as orange. Note that in both cases, binding of these proteins to IPMK would be predicted to sterically interfere with IPMK kinase activity.
